# Unveiling dengue fever prevention and control strategy in Ethiopia: a policy landscape analysis

**DOI:** 10.11604/pamj.2025.51.42.47457

**Published:** 2025-06-11

**Authors:** Chalachew Sisay, Netsanet Worku, Tadesse Awoke, Kassahun Alemu

**Affiliations:** 1 Institute of Public Health, College of Medicine and Health Sciences, University of Gondar, Gondar, Ethiopia,; 2 Ethiopian Public Health Institute, Addis Ababa, Ethiopia,; 3 Department of Human Nutrition, Institute of Public Health, College of Medicine and Health Sciences, University of Gondar, Gondar, Ethiopia,; 4 Department of Epidemiology and Biostatistics, Institute of Public Health, College of Medicine and Health Sciences, University of Gondar, Gondar, Ethiopia

**Keywords:** Dengue, policy analysis, prevention and control, Ethiopia

## Abstract

**Introduction:**

dengue fever is an emerging mosquito-borne viral disease in Ethiopia. To sustainably address its incidence and impact, a strong policy framework is essential for enhancing prevention and control efforts. This study aimed to explore existing operational policies and identify the facilitators and barriers to implementing tailored, country-specific strategies.

**Methods:**

the study employed a mixed-methods design. A desk review analysis, utilizing the policy triangle framework, examined existing operational policy documents, analyzing their content, context, actors, and processes. Additionally, eight key informant interviews were conducted with stakeholders, leading to the development of a thematic analysis based on the study objectives.

**Results:**

six dengue fever prevention and control policy documents were identified. Two from the Ministry of Health and four from the Ethiopian Public Health Institute lacked details regarding target populations, geographic areas, risk levels, and the focus of interventions across different contexts. A qualitative review revealed that the lack of tailored, country-specific intervention strategies, the absence of dedicated programs, and insufficient budget allocation to dengue fever prevention and control. Furthermore, collaboration among ministries and health stakeholders was poor, and the application of operational research was limited.

**Conclusion:**

the identified gaps emphasize the urgent need for a comprehensive strategy to address dengue prevention and control. This involves developing tailored, country-specific intervention strategies and establishing a dedicated dengue program with sufficient resources. Robust early warning surveillance systems, coupled with strengthened intra- and inter-sectoral collaboration, are crucial for coordinated dengue fever control. Implementing cost-effective, locally adapted integrated vector management strategies, guided by a one health approach.

## Introduction

Dengue fever (DF) is a systemic and dynamic viral infection transmitted between humans by the primary vector, *Aedes aegypti* mosquitoes, during daytime bites [1,2]. The infection is caused by any of the four distinct serotypes (dengue virus 1-4) of single-stranded RNA viruses [3]. Individuals are at greatest risk for severe dengue when they experience two sequential infections with two different serotypes within 18 months [4].

The incidence of dengue in the African continent has surged. All four dengue serotypes are present, and 47 of 55 African countries have reported the *Aedes aegypti*. Additionally, 34 countries have documented DF cases [5].

Ethiopia's first major DF outbreak happened in Dire Dawa City, with 11,409 cases reported [6]. Outbreaks also occurred in Dire Dawa (2014, 2016), Godey Town (Somali Region, Eastern Ethiopia), and Ada'ar Woreda (Afar Region, Northeastern Ethiopia) [7-9]. During the COVID-19 pandemic response, the outbreak continued in Dire Dawa City, the Warder Town of the Ethiopian Somali region, and Gewane District, Afar Region [10-12]. The 2023 outbreak started in Mille, Afar, affecting seven nearby towns in Afar, as well as Dire Dawa, Methara in central Oromia, and several districts in the Somali Region of Ethiopia [13-17].

To address the health impacts of dengue fever, the WHO has launched the initiative titled “Ending the Neglect to Attain the Sustainable Development Goals: A Roadmap for Neglected Tropical Diseases (NTDs) 2021-2030”. This framework recognizes dengue as a neglected tropical disease and aims to reduce the dengue case fatality rate to zero by 2030 [18]. In alignment with this initiative, many countries, including Ethiopia, are adopting these strategies to combat dengue. The Federal Ministry of Health (FMoH) of Ethiopia has integrated DF into its efforts against 12 other NTDs, aligning with the country's. Third NTD Master Plan (2021-2025) [19] and the Second Health Sector Transformation Plan (HSTP II 2021-2025) [20].

The Ethiopian Public Health Institute (EPHI) has worked with partners to combat increasing DF epidemics by implementing epidemic response activities in high-risk areas. These efforts included developing arboviral surveillance and response guidelines, developing epidemic plans, mandating immediate case reporting, and establishing sentinel surveillance sites. Despite these actions, DF prevalence and severity have risen, and existing prevention and control policies have not been adequately reviewed in the previous study. Therefore, it is unclear whether the Federal MoH and its implementing agencies, including EPHI, have effectively developed and executed a tailored, country-specific policy framework for dengue fever prevention and control. Furthermore, the robustness of current surveillance systems and early warning systems has not been adequately evaluated. Collaboration among stakeholders and different ministries in preventing and controlling dengue fever also requires further assessment. Addressing these gaps is essential for improving national dengue fever prevention and control efforts.

Therefore, the objectives of this study were to: 1) describe the existing operational policies of the Federal MoH and its implementing agencies related to DF prevention and control; 2) identify the key facilitators and barriers impacting the effective implementation DF prevention and control policy, and develop evidence-based recommendations for enhancing their application; 3) assess the functionality and effectiveness of current DF surveillance systems and inter-ministerial/stakeholder collaboration in the context of prevention and control strategies.

## Methods

**Study design:** this study adopted a mixed-methods design, strategically integrating desk review and qualitative approaches across all phases of data collection and analysis to comprehensively examine the policy landscape of dengue prevention and control. This methodological triangulation was employed to enhance the rigor, credibility, and trustworthiness of the findings. Recognizing that quantitative data alone could not fully elucidate the complexities of policy implementation and stakeholder engagement, qualitative methods were incorporated to provide richer contextual understanding, explore stakeholder perspectives, and elucidate the practical realities of dengue fever prevention and control strategies in the country.

**Setting:** Ethiopia's decentralized health system operates with the FMoH setting policy, Regional Health Bureaus (RHBs) adapt and implement these national policies within their specific regional contexts, while the EPHI provides evidence and coordinates emergency response [21]. A single confirmed case of DF is an immediately reportable disease. A majority of epidemic cases had been reported from the Afar, Dire Dawa, and Somali Regions (sub-national) of the country. The hot, arid climates common in these areas, combined with their geographic location bordering multiple East African countries and along existing trade routes, likely result in environmental conditions that allow *Aedes* mosquito populations to thrive.

The study participants were purposively selected for key informant interviews (KIIs) from RHB and Regional Public Health Institutes. In addition to these individuals, we included NTD and Public Health Emergency Management (PHEM) coordinators and officers working at national and internationally, who offered expert insights on how current dengue prevention and control policies were being put into practice at both national and regional levels. These staff members were actively involved in NTD program design, strategic planning, and implementation, as well as arboviral disease epidemic preparedness and response activities throughout the interview process.

**Data collection:** operational policy documents were gathered from various sources, including institutional websites and search engines. Key sources included the FMoH and EPHI websites, which the government and partners currently utilize for program purposes and epidemic response, serving as the selection criteria. Additional search was conducted on Google Scholar and through combinations of key terms such as “arboviral disease” or “NTDs master plan” or “climate-sensitive disease” or “vector surveillance” or “dengue plan” or “climate-sensitive disease” or “dengue guidelines”. Certain documents not available online were collected physically from organizations.

Following the ethical approval from the IRB, the principal investigator contacted eight respondents via phone and email to explain the study's purpose and schedule the interviews. Three respondents were interviewed face-to-face, while the remaining five were interviewed virtually and by phone. Prior to each interview, written informed consent was obtained from the participants interviewed in person, while verbal consent was recorded for those interviewed virtually and by phone. The investigator provided detailed information regarding their participation, the assurance of confidentiality, and their right to withdraw from the study at any time. All interviews were audio-recorded to ensure accurate data capture and transcription.

The data collection protocol was adapted and customized from a previous study focused on dengue fever policy [9,10]. The interviews ranged in duration from 25 to 55 minutes, with an average length of 40 minutes. Participants were interviewed using a semi-structured interview guide that covered four distinct thematic areas: (1) the availability of operational policies and strategies; (2) facilitators and barriers to key impacting the effective implementation; (3) evidence-based recommendations; and (4) functionality and effectiveness of current DF surveillance systems and inter-ministerial collaboration.

**Data analysis:** policy triangle framework: the framework was designed in 1994 by Walt and Gilson for the analysis of health sector policies. In 2008, Walt and colleagues made some slight changes to this framework. This model covers four general areas: content, context, actors, and policy-making process. Content includes policy objectives, operational policies, etc. Actors refer to acting and influential individuals and organizations. Context refers to social, economic, political, cultural, and other environmental conditions. The process includes four sections: agenda setting, policy formulation, policy implementation, and policy evaluation [22].

Policy documents related to the prevention and control of dengue fever were first collected from various websites. Following this collection, the study provided a summary of these documents, identifying their objectives, target populations, geographic areas, focuses of intervention, and relevance to DF. Subsequently, these policies were analyzed using the policy triangle framework. The next phase of the study involved conducting explanatory sequential KIIs with the individuals participating in the study. The KIIs sought to clarify and resolve any questions left unanswered by the initial desk review of policy documents.

Qualitative data from the KIIs were coded both manually and using the ATLAS.ti 9 computer program. The study adopted a systematic approach of reducing the qualitative data and categorizing it based on the study's themes. After analyzing the texts for recurring ideas, we identified significant findings. These results are presented as a combination of summarized meanings and direct quotations from the texts, which serve as evidence for our study's conclusions.

**Ethical consideration:** the study received ethical approval from the Institutional Review Board (IRB) of the College of Medicine and Health Sciences at the University of Gondar (Version: 12^th^ February 2024). Throughout the entire study, anonymity and confidentiality were maintained. Respondents were assigned unique identifiers.

## Results

**Descriptive analysis:** a total of six operational policy documents were identified from various organizations, as shown in Table 1. These documents originated from the FMoH (2 documents) and EPHI (4 documents). The review included three strategic plans, two guidelines, and one implementation manual. We reviewed these documents, considering their objectives, intended recipients, geographical scope, focus of interventions, and relevance to DF. The participants of KIIs involved eight public health experts (Table 2). Five with master's, two with doctoral, and one with bachelor's degrees, primarily from EPHI and MOH, with 9-22 years of experience. One interview was excluded due to data saturation.

**Table 1:** summary of Dengue-related operational policy documents by organization, objectives, target population, and geographic areas

S.no	Policy documents	Organization	Summary of objective	Target population	Geographic area	Intervention area (clinical, vector control, surveillance	Relevance to DF (Dengue fever)
1	Health sector transformation plan II (HSTP II) 2020/21-2024/25	MOH*	Strengthen measures to prevent/control Dengue and enhance health system resilience	Vulnerable populations, especially in urban areas	Across all regions of Ethiopia	Surveillance was a key strategy for malaria prevention and control, but the document did not clearly address its application for Dengue fever	The strategic document prioritized NTDs like schistosomiasis and lymphatic filariasis over common arboviral diseases such as Dengue
2	The third strategic planning and management (SPM-III) 2020/21-2029/30	EPHI*	Enhance Dengue management through improved outbreak response and diagnostics	Vulnerable groups in Ethiopia	High-risk regions in Ethiopia	The document does not include prevention strategies such as vector control, surveillance, or clinical management	Generally, includes a framework for public health emergency surveillance and response, emphasizing coordination and collaboration, but it notably lacks a clear focus on these aspects specifically for Dengue fever prevention and control
3	The third national neglected tropical diseases strategic plan, 2021-2025	MOH	Control Dengue as part of the neglected tropical diseases strategy	Communities at risk, particularly vulnerable groups	Regions with endemic Dengue in Ethiopia	The strategic plan identifies stakeholder roles simplistically but lacks clear surveillance strategies, raising concerns, and insecticide-treated bed nets (LLINs), indoor residual spraying (IRS), and breeding site control are proposed for Dengue prevention, strategies like space sprays, larviciding, traps	The strategic document identified stakeholders, partners (including the community), community awareness strategies, and strong coordination initiatives. However, it did not specify the target population for Dengue or chikungunya community awareness efforts
4	Arboviral diseases surveillance and response guideline, February 2022	EPHI	Guide surveillance and control of Dengue fever to reduce health impacts	At-risk communities and health professionals	Dire Dawa Afar Region Somali Region	During pre-epidemic periods, this guideline recommends sentinel and event-based surveillance. In epidemic situations, it suggests using a combination of passive, active, or community-based surveillance, specifically targeting populations in epidemic-prone areas	The guideline prepared general prevention and control measures for arboviral diseases across the country, but it is not specific to DF
5	Climate sensitive diseases surveillance and early warning system implementation manual in Ethiopia, June 2022	EPHI	Strengthen early warning systems for Dengue to enhance public health responses	Vulnerable groups, particularly children and the elderly	Urban and peri-urban regions in Ethiopia	The manual review indicates the use and recommendation of a sentinel surveillance system for early outbreak detection in epidemic-prone areas.	The implementation manual details public health awareness strategies but lacks specific guidance for Dengue fever prevention
6	Arboviral disease vectors surveillance and control guideline, October 2021	EPHI	Guide surveillance of Aedes mosquitoes to reduce Dengue risk	Residents in urban high-risk areas	Regions in Ethiopia prone to Dengue outbreaks	Arboviral vector control methods are not specific to DF	Arboviral vector control methods are not specific to DF

MOH: ministry of health; EPHI: Ethiopian Public Health Institute

**Table 2:** demographics characteristics of participants in dengue fever policy landscape analysis (February 20 - April 15, 2024)

Participant ID	Educational level	Institution	Position/role	Years of experience
P1	Master's degree	DD RHB *	Regional Public Health Emergency Management (PHEM) 1	13
P2	Master's degree	EPHI	National Public Health Emergency Management (PHEM) 1	13
P3	Master's degree	EPHI	National Public Health Entomologist	15
P4	Doctoral degree (Ph.D.)*	IHA *	Program expert 1	22
P5	Bachelor’s degree	EPHI	National Public Health Emergency Management (PHEM) 2	14
P6	Master's degree	APHRI*	Public Health Emergency Management	9
P7	Doctoral degree (Ph.D.)*	MOH	Program expert 1	20
P8	Master's degree	MOH	Program expert 2	9

Ph.D: doctor of philosophy; DDRHB: Dira Dawa Regional Health Bureau; IHA: International Health Agency; APHR: Afar Public Health Research Institute

### Actors

**Stakeholder role and collaboration:** the national arboviral surveillance and response and NTD master plans emphasized and incorporated broader disease coverage and stakeholder lists. However, they didn't clearly define the roles and collaborative efforts of stakeholders in terms of response and program strategies. This was echoed with: “*stakeholder collaboration for dengue fever prevention and control is inadequate, with the health sector primarily shouldering its impacts. While initiatives such as university collaborations during outbreaks offer some hope*” (interviewee 6).

The reviewed policy document lacked clear definitions of community roles and responsibilities and also failed to specify intervention measures for household, community, and institutional settings. Participants consistently highlighted a lack of institutional collaboration. Two respondents from RHB (interviewee 1 and 6) noted: “*the health sector was fully accountable for preventing and controlling dengue fever. However, collaboration between local universities and city administrations was minimal and fragmented, primarily focusing on vector control rather than a comprehensive prevention strategy*”.

The FMoH, through its HSTP-II (2020/21-2024/25), emphasizes that engaging community members in vector-borne disease prevention and control efforts to fosters ownership and sustainability; program expert 2 (interviewee 8) at the FMoH clearly stated this: “*empowering the community and improving living standards are crucial for preventing vector-borne diseases. This can be achieved by enhancing personal hygiene and sanitation, promoting awareness of preventive measures, and encouraging community participation in vector control activities to create a healthier environment*”.

The EPHI developed the third Strategic Planning and Management (SPM-III) document, a ten-year plan. This policy document strongly suggests initiatives for epidemic prevention and control of dengue fever, including the establishment of a task force for curbing epidemics. The respondents (interviewee 2) highlighted and suggested that: “*at the national level, it is important to develop a national task force and advisory group comprising representatives from various organizations, including research institutes, experts, and universities in affected areas*”.

The FMoH-NTDs master plan lacked sufficient detailed evidence on specific areas and risk populations, focused intervention, and the distinct roles of different government stakeholders. However, the proposed intervention for tackling dengue prevention and control suggests using approaches already employed in malaria prevention and control efforts. One of the MOH program officers 2 (interviewee 7): “*controlling dengue fever requires collaborative efforts and adaptive interventions beyond malaria vector control… Key strategies include enhancing community engagement, improving early detection in high-risk areas, and researching climate impacts on the disease…a multi-sectoral approach involving diverse stakeholders is essential for effective prevention and control*”.

### Content

**Identified operational policy and gaps:** the policy documents that were reviewed related to DF in the country included: The Federal MoH's (HSTP-II 2020/21-2024/25), the EPHI's (SPM-III 2020/21-2029/30), and the third NTD Strategic Master Plan (2021-2025). National Arboviral Disease Surveillance and Response guidelines (2022), the Arboviral Disease Vectors Surveillance and Control Guideline (2022), and the Climate Sensitive Diseases Surveillance and Early Warning System Manual (2021) (Table 1).

The lack of a specific national strategy for dengue fever prevention and control was a key finding of the desk review and qualitative study. This absence was noted despite the presence of broader national health frameworks like HSTP II and the NTDs' third Master Plan. Seven interview participants (P1, P2, P3, P4, P5, P6, and P8) specifically pointed out this gap.

The EPHI-PHEM center has been actively involved in early warning, surveillance, and response to public health emergencies, including dengue fever. In 2021, it developed national guidelines for arboviral surveillance and response to address outbreaks, including dengue fever. However, these guidelines do not adequately focus on the urgent needs for severe dengue clinical management and lack tailored strategies for dengue prevention and control. Respondent from EPHI (interviewee 5) highlighted that: “*the guidelines are lacking in scientific evidence, especially in the outdated and generic case management section. The inclusion of multiple arboviral diseases…hinders a focused approach to dengue fever and overlooks critical regions and diverse populations in the country*”.

In resource-limited county like Ethiopia, an integrated approach to managing acute febrile illnesses is vital for more accurate diagnoses, efficient use of resources, and better patient care; respondent from IHA (interviewee 4) emphasized; “*the need to enhance quality assurance, care, and diagnostic tests…recommended integrating case management for diseases with similar febrile symptoms to dengue fever, such as malaria and chikungunya, in line with WHO recommendations. Ruling out these conditions would improve case management, reduce resource use, and increase overall effectiveness*”. Respondent (interviewee 2) emphasized that using evidence and research (operational research uptake) is crucial when developing dengue prevention and control strategies. “A nationwide sero-survey is needed to evaluate differences between urban and rural areas for effective intervention strategies. The country should adopt successful strategies from other nations through experience sharing and utilize local research that reflects its context, including urbanization”.

Over the past decade, the public health impact of DF has significantly escalated in the eastern and northeastern regions of the country. However, current policy documents fail to provide alternative prevention and control measures specific to dengue. Instead, they rely solely on strategies adopted from the NTDs master plan, such as case management, surveillance, and vector control, which are drawn from the WHO-NTD master plan 2021-2025. This approach overlooks the unique epidemiological characteristics of dengue and the critical need to tailor interventions to the local context. However, two respondents (interviewees 2 and 7) emphasized the necessity of implementing a “One Health” approach in epidemic-affected areas.

Arboviral Disease Vectors Surveillance and Control Guideline (2021) and National Arboviral Disease Surveillance and Response guidelines (2022) outline the main intervention strategies for both adult and larval vector control, emphasizing the application of various chemicals at potential breeding sites but an EPHI Entomologist (interviewee 3) points out that these guidelines don't take the country's economic situation into account: “*the challenge in the prevention of dengue vector outdoors, most interventions in our country focus on indoor settings* (manly for the prevention of malaria) *…collaboration with other sectors on urbanization and environmental health is vital …by adopting an integrated vector management approach*”.

### Context

**Leadership and decision making:** federal MoH faces complex health priorities, demanding careful resource allocation and strategic decisions to meet Ethiopia's diverse health needs, as emphasized by a MOH program expert in (interviewee 8): “*dengue is an emerging public health concern in the country… with no previous national program to address it …challenges include a lack of understanding of the disease burden… absence of a specific strategy… inadequate budget allocation for program and operational research…and poor early detection at health facilities, often misdiagnosed as other febrile illnesses*”.

Another respondent from EPHI (interviewee 2) expressed this concern during the interview: “*there is currently no specific national strategy or policy for comprehensively controlling and preventing dengue fever in the country. Efforts have been largely reactive, responding only after outbreaks occur, rather than proactive… At the strategic level, including the Ministry of Health, there is no dedicated program for dengue management*”. The Arboviral Disease Surveillance and Response guidelines (2022), document mentions proposed dengue surveillance methods, like sentinel sites for early outbreak detection and response. However, it notes the absence of a standard online or offline platform for real-time dengue case data collection and analysis.

An effective early warning surveillance system is critical for the timely detection and rapid response to DF epidemics, as well as for sustainable program implementation at all levels of the healthcare system. A significant gap in effective detection and response often stems from inadequate infrastructure and the underutilization of digital health systems, particularly in resource-limited settings. Four study participants (P1, P4, P5, and P6) indicated that in areas with poor infrastructure development: “*the government's establishment of an offline mobile-based dengue surveillance platform due to the poor infrastructure in the county. They underscored that this offline system would be effective in areas where the internet is unavailable*”.

### Process

**Policy implementation:** the government led a collaborative, top-down process to design operational policy documents, with the WHO, UNICEF, and US CDC primarily offering final technical assistance for community-level implementation. The HSTP-II and SPM-III, developed every five and ten years, respectively, receive financial support from international organizations (including WHO, UNICEF, US CDC) and government contributions. Implementing health policies in Ethiopia follows a top-down structure, but challenges arise from decision-makers' perceptions and vulnerabilities, potentially due to insufficient incorporation of strong scientific evidence and competing public health priorities. The qualitative study also revealed a heavy reliance on donor funding for national programs, with arboviral diseases receiving limited financial support, as highlighted by a participant (P8): “*there is no specific budget line within the NTD department, and I'm not sure*”. One of the respondents from the Ministry of Health (interviewee 8) discussed the challenges in the implementation of the dengue fever prevention and control policy: “*there is a lack of understanding of the disease burden among senior management… the exclusion of the dengue program in senior management decisions*”.

Two respondents from RHB and regional public health institute (interviewees 1 and 6), although they are not aware of the national NTD program, emphasized the implementation of existing operational policy for dengue fever: “*the higher officials are aware of the condition of the disease as it has caused many officials to fall ill… Their response is great*”. While lower-level health sector cadres are heavily involved in DF epidemic response activities, this could result in top-level health cadres becoming less engaged in understanding epidemic dynamics. As a result, the epidemics, particularly those concentrated in the eastern part of the country, might give the necessary policy attention and agenda prioritization.

## Discussion

While a desk review identified six operational strategy documents related to DF, a critical gap exists due to the absence of a specific policy dedicated to dengue prevention and control in the country. The reviewed documents lack the necessary specific details for effective implementation, including clearly defined target populations, geographic areas, and detailed intervention plans across the health system and community levels. Key informant interviews revealed significant gaps hindering effective DF prevention and control. These are the lack of a national program and insufficient resources, and low political attention at the FMoH. Furthermore, the non-existence of a national technical advisory group, task force to guide evidence-based interventions of epidemics, weak inter- and intra-sectoral collaboration, and a lack of proactive early warning surveillance mechanisms were consistently identified.

In Ethiopia, the strategy document lists several organizations from non-health (inter) sectors involved in DF prevention and control. However, the collaborative efforts in dengue prevention and control have not been strong and have not yielded the intended results. The concept of intersectoral collaboration action for health promoted by WHO [16], aims to collaboratively address issues to achieve health outcomes that are more effective, efficient, and sustainable. This is similar to a study conducted in Mexico highlights that the legal framework calls for an intersectoral response to the dengue epidemic. However, the burden of activities in terms of financial and human resources tends to fall on local health services [23]. This issue is not unique to Ethiopia. The key informant interviews highlighted a notable absence of robust collaboration between the FMoH) and the EPHI. Despite policy documentation outlining data sharing protocols, a study participant (interviewee 2) stressed that a lack of cohesive collaboration and coordination within these organizations resulted in inefficiencies and missed opportunities for integrating strategies to prevent DF.

In the Philippines, counties have proactively established a nationwide dengue prevention and control program, demonstrating a significantly greater emphasis on addressing the disease. This program, with its focus on integrated vector control, clearly defined goals and strategies, and emphasis on community involvement [24]. In contrast, the Federal MoH appears to prioritize malaria and other NTDs over dengue, as evidenced by the absence of a dedicated program, a technical advisory group, clearly defined goals, sufficient community involvement strategies, and a national task force for dengue. This analysis underscores a notable disparity in preparedness when compared to the Philippines' established, nationwide dengue program.

The selection of intervention strategies is often depending upon available evidence and resources. In Bangladesh and China, for instance, a 'One Health' approach is advocated by the government, emphasizing the critical interconnectedness of the disease, its vectors, and their ecological environment [25,26]. Although no policy document suggested this approach, two respondents, one from the FMoH (interviewee 7) and the other from the EPHI (Interviewee 2), independently recommended implementing the 'One Health' approach to substantially reduce the incidence of dengue epidemics and enhance overall prevention and control efforts within the country.

The policy documents indicate an increasing burden of dengue fever in urban Ethiopia, underscoring the need for effective interventions. An entomologist (interviewee 3) recommended implementing integrated vector management (IVM) in these areas, which the WHO supports as a resource-limited and sustainable approach. IVM focuses on capacity building, enhanced surveillance, and cross-sector coordination [27]. By adopting and incorporating IVM principles into health policy, Ethiopia could enhance its epidemic response to DF and reduce the overall burden of vector-borne diseases, and it could strengthen urban DF impacts.

In Sri Lanka, the recurrent outbreaks of dengue prompted the Ministry of Health to establish a dedicated national dengue control program to coordinate integrated vector management efforts. Additionally, the development of a presidential task force marked a significant turning point in the country´s dengue control program [28]. Similarly, in Singapore, not only is the national dengue program being enhanced, but inter-epidemic surveillance and control are also established and strengthened by legislation [29]. Recognizing Ethiopia's diverse geographic, topographic, political, and socioeconomic contexts is crucial for significantly enhancing the dengue prevention and control strategy. Establishing a dedicated national dengue program with political will would improve accountability and responsibility in program management and the curbing of DF epidemics.

To effectively combat dengue in Ethiopia, the country should learn from countries with significant experience in this area, understanding that strong, dedicated experts and community engagement are essential for success. For example, Thailand's 3-3-1 strategy, a community-based effort, requires dengue cases to be reported within three hours, the patient's home to be cleared of infected mosquitoes within three hours, and insecticide spraying within a 100-meter radius to occur within one day [30]. Through the Ministry of Health's initiatives, Indonesia is making significant progress in DF prevention and control. These initiatives involve establishing legal frameworks for program implementation and environmental standards, alongside fostering partnerships to decentralize governance [31]. In Ethiopia, dengue categorizes it as one of the NTDs and an immediately reportable disease [6,22]. However, dengue prevention and control are currently inadequate and fragmented, hindered by inconsistent support and potentially poor resource allocation, despite the growing disease burden.

A review of the existing national policy for Ethiopia reveals that it doesn't clearly outline the role of individuals and community participation in addressing dengue fever within epidemic-affected areas. In contrast, South American countries have successfully employed the Integrated Management Strategy for dengue Prevention and Control (IMS-dengue) aimed at strengthening national dengue programs and emphasizing individual and community behaviors and diminishing the risk factors for transmission through coordinated measures inside and outside the health sector [32].

Eastern and northeastern regions of Ethiopia have experienced a surge in severe DF, resulting in a high number of deaths. This increase is due to frequent outbreaks and the introduction of different serotypes [13]. Furthermore, existing operational policy documents on arboviral disease surveillance and response guidelines do not incorporate best practices from the international dengue case management guideline [33]. While the HSTP II [20] includes a strategy to train health professionals in various specialty programs, but it lacks a plan to train specialists in infectious diseases, either domestically or internationally.

Ethiopia has experienced a notable shift in its urban demographic since the turn of the millennium. Between 2000 and the present, the urban population has grown from 15.5% to 20.2%, and projections suggest a potential tripling of this figure by 2037 [34,35]. In contrast to this significant and ongoing urbanization, and the associated increase in the scale and seriousness of DF within urban areas, the reviewed policy documents fail to clearly outline specific mitigation strategies tailored to these urban environments.

Ethiopia's legal surveillance framework requires immediate reporting of DF cases, from point-of-care to district PHEM in 30 minutes and then to national PHEM within 2 hours [36]. Despite this established system, the country lacks a standard surveillance data platform for real-time dengue case data collection and analysis. In contrast, countries endemic to dengue, such as China [37] and Brazil [38], are implementing online multi-level disease surveillance networks at both national and local levels, allowing for real-time collection and analysis of dengue case data and entomological data. This enables a swift response in the early stages of outbreaks.

To effectively address the increasing impact of DF in Ethiopia, FMoH must prioritize urgent and comprehensive action targeting vulnerable urban and underserved populations. To effectively mitigate this growing threat, a holistic approach is essential. This includes developing a tailored and country-specific strategy and establishing a dedicated DF intervention plan, distinct from broader NTD strategies. In addition, establishing an adequately funded national dengue program with an active national technical advisory group is crucial and enhance collaboration and coordination. Countries must invest in context-specific early warning surveillance platforms that incorporate both offline and real-time data collection capabilities.

## Conclusion

The burden of DF is demonstrably increasing in the country; this study highlights critical weaknesses hindering effective control and prevention efforts. We found a significant lack of a dedicated national dengue strategy, limited inter- and intra-sectoral collaboration, and poor community engagement approach, and no well-funded national program. Policy documents lack clear targets and localized strategies, and the national response is largely reactive due to poor early warning, absent real-time surveillance, and low research uptake. To effectively address the rising incidence of dengue fever, the FMoH, in close collaboration with RHBs, must urgently prioritize the development and implementation of a comprehensive, tailored, and country-specific national dengue strategy. Furthermore, the FMoH should establish a national dengue technical advisory group and a well-funded dengue program and community engagement strategy, establish a robust early warning and enhance proactive surveillance system, and promote operational research. Simultaneously, regional health bureaus should be empowered to localize response strategies. However, current policy analysis is limited by its focus on national documents, excluding regional documents and non-health sector perspectives, highlighting the need for broader future research.

### What is known about this topic

- Existing arboviral surveillance and response guidelines, including those for dengue fever, have been developed by partners and stakeholders to address epidemic control;
- Ethiopia has committed to and is currently implementing the World Health Organization's “end neglected tropical diseases 2030 goals”, which encompass dengue fever control;
- Early warning surveillance systems in the country are often characterized by a reactive approach and inadequate performance, revealing gaps in coordination and preparedness.

### What this study adds

- The study demonstrates the urgent need for country-specific dengue strategies (policy) tailored to and country-specific;
- This study highlights the critical importance of establishing dengue programs or units with adequate budgets at all administrative levels for effective disease control;
- This study underscores the necessity of implementing proactive and robust early warning surveillance systems and strengthened coordination mechanisms for timely and effective dengue outbreak response.
